# Supplementary material for: Epidemiology of Echinococcus granulosus sensu lato in the Greater Horn of Africa: A systematic review
Source: PLoS Negl Trop Dis. 2024 Jan 25;18(1):e0011894. doi: 10.1371/journal.pntd.0011894 (PMC10810510; doi:10.1371/journal.pntd.0011894)
Supplement: S2 Checklist — (DOCX) [file pntd.0011894.s002.docx]

**S2 Checklist. A template designed for consulting experts for grey literature.**

**Epidemiology of *Echinococcus granulosus* infection in the Greater Horn of Africa**

Dear colleague,

We are currently looking into the epidemiology of echinococcosis in humans and animals in the Greater Horn of Africa by means of a systematic review. The list of countries identified for the review are **Eritrea, Ethiopia, Djibouti, Kenya, Somalia, South Sudan, Sudan, Tanzania and Uganda.**

In order to easily access "sleeping data", we would kindly like to ask you to fill out the tables below to help us **facilitating the search** and **gain access to databases unknown to us**.

We would like to acknowledge everyone who offered an information sources or data.

We are very grateful for your precious help.

Thank you,

Team members of the review

# **National journals or Epidemiological bulletins**

Please provide a list of national or regional journals that may contain information on human and/or animal echinococcosis in the Greater Horn of Africa. Feel free to use as many rows as needed.

| **Name of Journal or Epidemiological bulletin** | **Country/region** | **URL (if available)** |
| --- | --- | --- |
|  |  |  |
|  |  |  |
|  |  |  |

# **MSc/PhD dissertation databases**

Please provide a list of MSc/PhD dissertation databases that may contain information on human and/or animal echinococcosis in the Greater Horn of Africa. Feel free to use as many rows as needed.

| **University** | **Country** | **URL** |
| --- | --- | --- |
|  |  |  |
|  |  |  |
|  |  |  |
|  |  |  |

# **National institutes**

Please provide the names and websites of the national institutes that are responsible for the concerned activities. Feel free to duplicate rows if needed.

| **Activity** | **Country** | **Institute/Network** | **URL** |
| --- | --- | --- | --- |
|  |  |  |  |
|  |  |  |  |
|  |  |  |  |

# **Conference proceedings**

Please provide the names of professional organizations that have conferences at which researchers might be presenting work related to echinococcosis in the Greater Horn of Africa

| **Conference name** | **URL (if available)** |
| --- | --- |
|  |  |
|  |  |
|  |  |
|  |  |

# **Suggested expert(s) in the field**

Please provide name of your suggested key author/s and expert/s who can be a valuable source of information about echinococcosis in the Greater Horn of Africa.

| **Author or expert name** | **Country** | **Contact address** |
| --- | --- | --- |
|  |  |  |
|  |  |  |
|  |  |  |

# **Other sources (If available)**

Please provide articles accepted for publication or in press, grey literature documents, as well as sources of grey literature they you feel would be important to include in the review.

| **Title of article or name of grey literature source** | **Country** | **Status or URL (if available)** |
| --- | --- | --- |
|  |  |  |
|  |  |  |
|  |  |  |
|  |  |  |
