# Supplementary material for: Epidemiology of Echinococcus granulosus sensu lato in the Greater Horn of Africa: A systematic review
Source: PLoS Negl Trop Dis. 2024 Jan 25;18(1):e0011894. doi: 10.1371/journal.pntd.0011894 (PMC10810510; doi:10.1371/journal.pntd.0011894)
Supplement: Attachment — Submitted filename: Response to Reviewers _PNTD-D-23-00636_Final.docx [file pntd.0011894.s005.docx]

**Response to Reviewer Comments - Manuscript ID PNTD-D-23-00636**

Dear Editors,

Dear Reviewers,

We would like to express our sincere gratitude for dedicating your time and expertise to thoroughly review our manuscript, "Epidemiology of *Echinococcus granulosus sensu lato* in the Greater Horn of Africa: A systematic review," submitted to PLoS Neglected Tropical Diseases. The constructive comments have significantly improved the manuscript. Below we have addressed each comment. Line numbers in our responses correspond to the revised document with track changes, and hence they might be subjected to change in the clean document.

Best regards,

Weldegebrial Aregawi.

**Reviewer #1:**

**This paper is well written realizing a systematic review of the data about *E. granulosus sensu lato* in a specific area of Africa.
The introduction gave a clear overview of the state of the art for CE in GHA. The protocol of the systematic review is relevant, well described and properly applied. The results are interesting and discussed in a relevant way, being aware of the limitations of the study due to the data obtained.**

We thank the reviewer for the positive feedback on our manuscript.

**I have only some minor comments for this very interesting paper.**

**l.90: close the brackets after Uganda**

These brackets have been closed as suggested on line 90.

**l. 94: please also close the brackets**

These brackets have been closed on line 94.

**Regarding species/genotype identification for both humans and animals, it may be interesting to specify the different methods used especially to confirm (or not) the high specificity.**

We have now included the different methods used for humans (lines 243-245), intermediate host (lines 325-327) and final hosts (line 404).

**l. 293-303: no case of E. ortleppi in humans? As it is known as zoonotic, it may be interest to briefly discuss this absence of report even some cases probably exist.**

Indeed, we did not find any report of *E. ortleppi*, and this has now been made explicit in the result section (lines 319). Considering that there are only few reports of human infections caused by *E. ortleppi* in other parts of the world [1] and the absence of *E. ortleppi* in our study we did not further discuss this observation.

**l. 331-332: poor or medium BCS is observed infected animals, but is it considered as a consequence of CE or a risk factor to develop infection?**

The direction of association between poor or medium Body Condition Score (BCS) in animals with cystic echinococcosis (CE) is not uniformly established in literature. Notably, studies included in our analysis, such as [2–9], align with the findings of an earlier study by Polydorous [10]. Polydorous [10] suggested that poor body condition could result from infection with a high number of cysts, leading to retarded performance and growth.

Conversely, other studies like [11,12], indicate that well-fed animals with good body condition may resist or limit the severity of CE, while poorly nourished animals may be less competent in fighting against infections.

Overall, this question about causality, we believe, is beyond the scope of the current review. Therefore, we have opted not to change the manuscript.

**l. 337-339: as the organ most frequently infected is not the same regarding the intermediate host species, is it possible to combine with molecular identification of the CE species to confirm or not the existing data?
The same question regarding fertility among studies, which can provide both data (species and fertility of cysts). The aim as for organs infected is to check if there are no differences with global observations.**

While we appreciate the suggestion, conducting this combined analysis was beyond the scope of our research objectives. Therefore, we have chosen not to change the manuscript. However, we would like to draw the reviewer's attention to relevant studies [13–17], that explored associations among cyst status (fertility), organ location, and genotype identification of *Echinococcus* spp. in different livestock species in our study area.

**l. 371: I suggest to rather used definitive than final for the title**

As suggested, we changed the title as per the suggestion (line 392) and we’ve adapted the term final host to definitive host throughout the rest of the entire document as well.

**l. 404: it is five species described not five genotypes**

This has been corrected accordingly in line 425 and throughout the entire document.

**l. 432-434: it would be interesting to compare the prevalence in humans in GHA with those observed in North-Africa which is known to be highly endemic with numerous data available.**

We have incorporated the prevalence comparison for Northern Africa from an Africa-wide systematic review study (lines 456).

**l. 455: please suppress the second "s" to obtain focused**

This has been corrected on line 480 as well as in lines 213 and 248.

**Maybe useful to indicate that a standardization or harmonization of the diagnostic tests would be also useful.**

We agree that standardization or harmonization of the diagnostic tests is important, but we do not see this as a stand-alone recommendation. Rather we see this as part of the need for more studies with appropriate study designs, in which harmonization of diagnostics across these studies is a must. We have therefore revised the sentence on line 517 to make this more explicit.

**Reviewer #2:**

**A systematic review of the literature on Echinococcus granulosus sensu lato species in Africa, as well as the rest of the world, is essential to advance knowledge, inform policy and implement effective control and prevention measures for echinococcosis, ultimately leading to improved global public health outcomes.**

**In the present study, a total of 980 records were retrieved from a literature search, of which 247 met the inclusion criteria for the systematic review. The majority of the records described data from Ethiopia, Kenya and Sudan. Quality and risk of bias were assessed for 177 population-based studies, with 40.7% of the studies being of high quality. The prevalence of CE in humans ranged from 0.33% to 3.0% based on ultrasonography and 6.5% based on serology. In animals, the prevalence ranged from 0.01% to 88.4% in cattle, 0 to 65.2% in goats, 0.05% to 10% in pigs and 0 to 67.8% in sheep. E. granulosus s.s. was the most common genotype found in humans and animals. The lack of well-designed studies and the wide range of diagnostic tools used make it difficult to accurately assess the epidemiology of CE in the Greater Horn of Africa.**

**Overall, the study was conducted and the data analysis and interpretation were performed in a good, transparent and understandable manner. The manuscript is well written and understandable. However, there are some points that should be at least partially improved regarding the epidemiological and statistical data analysis. See below for details:**

We thank the reviewer for the constructive comments that helped further improve the quality of the paper.

**1. What is generally missing is meta-analysis. When prevalence data from several studies are combined, this is the most common and powerful method. Meta-analysis combines estimates from individual studies to produce an overall summary estimate of prevalence, often using statistical techniques such as random-effects or fixed-effects models. Meta-analysis can provide a more precise and robust estimate of prevalence and can assess heterogeneity between studies.**

We appreciate the suggestion for a meta-analysis. While a systematic review can be supplemented with a meta-analysis, we have reservations about its applicability in our case. The substantial diversity in survey designs introduces challenges, resulting in wide confidence intervals and potential heterogeneity. This variability, combined with varying study quality, raises uncertainties about the unbiased nature of the estimates. Moreover, for the human host in particular, the number of studies is too small to justify a random-effects model. Given these considerations, we have deliberately focused on a systematic review, as reflected in the manuscript's title and objectives. We believe this approach ensures a comprehensive assessment of the available evidence in light of the study limitations.

**a. Forest plots would be useful to display the results of individual studies together with the pooled summary estimate from the meta-analysis. Forest plots allow you to visualise the variability between studies and to see the overall trend.**

We appreciate the suggestion regarding forest plots. However, in our case, we opted to include the calculated confidence intervals of the prevalence estimates for each study (Table 1, and S1 file, Tables S7 and S12). This approach effectively conveys the same information as forest plots, as the estimate and confidence interval together provide a comprehensive understanding of the study results. Including both forest plots and confidence intervals would essentially present duplicate information. We believe that emphasizing confidence intervals serves the purpose of conveying the variability between studies while avoiding redundancy. For our argumentation on why we opted to refrain from a meta-analysis, see the previous comment.

**b. Subgroup analysis, which is more detailed from a statistical point of view, would also provide clearer and possibly deeper insights if there are substantial differences between studies.**

We did not conduct any formal statistical subgroup analysis in our study for similar reasons as explained above.

**c. Meta-regression could also be done, because you have specific covariates (e.g. age, sex, study quality) that may affect the prevalence estimates, you can do a meta-regression to explore their potential effects.**

As indicated above, we did not conduct any additional analysis, including meta-regression. The decision was based on the diverse survey designs and the variability in study quality across the different studies, which could potentially impact the reliability and interpretation of the results.

**d. A cumulative meta-analysis might provide information on whether there is a trend in the prevalence data over time...**

As mentioned previously, we refrained from conducting additional analyses, including cumulative meta-analysis.

**2. Another suggestion would be to calculate the 95% confidence intervals and add them to the calculated prevalences (e.g. lines 338-339, Table 2).**

To avoid making the text too heavy due to the inclusion of 95%CIs throughout the manuscript, we have opted to only show the 95%CI for the reported prevalence estimates in each of the included population survey studies. As detailed in subsection 'a' above, we have provided 95%CIs for the included intermediate host studies in S7 Table and for the final host studies in S12 Table. In order to be consistent, we have now also added the 95%CI for the human host population survey studies in Table 1 at line 265.

However, it's important to note that in Table 2 and at various points in the text, proportions rather than prevalence estimates are presented. For these proportions, we have chosen not to provide 95%CIs to uphold consistency with our selective approach and to avoid complexity in the presentation of the data.

**3. Lines 330-331: Here it is not clear how significance was tested. This missing information should be added with appropriate p-values.**

The sentence in question has been rephrased to enhance clarity: 'Studies assessing associations between age and CE reported a higher prevalence in older animals' on lines 349-350. It's important to note that the described risk factors were risk factors presented as such in the respective records. In these studies, the significance of association was investigated using different techniques, including chi-square testing.

**References**

1.	Shi Y, Wan X, Wang Z, Li J, Jiang Z, Yang Y. First description of Echinococcus ortleppi infection in China. Parasit Vectors. 2019;12: 398. doi:10.1186/s13071-019-3653-y

2.	Debela E, Abdulahi B, Megersa B, Kumsa B, Abunna F, Sheferaw D, et al. Hydatidosis of camel (Camelus dromedarius) at Jijiga municipal abattoir, Eastern Ethiopia: prevalence, associated risk factors and financial implication. J Parasit Dis Off organ Indian Soc Parasitol. 2015;39: 730–735. doi:10.1007/s12639-014-0430-x

3.	Tadesse A, Negash N. Prevalence and economic connotation of bovine and caprine hydatidosis at Abergele International Export Slaughterhouse, Mekele, Tigray Region. Ethiop Vet J. 2022;26: 38–56. doi:10.4314/evj.v26i2.3

4.	Metages Y, Simegnew A, Mesfin S, Mesfin M. Prevalence and Associated Risk Factors of Hydatidosis in Cattle Slaughtered at Dejen Municipal Abattoir in Amhara Regional State , Ethiopia. 2020;28: 10–16. doi:10.5829/idosi.mejsr.2020.10.16

5.	Adane M, Guadu T. Bovine Hydatidosis: Occurrence, Economic and Public Health Importance in Gondar ELFORA Abattoir. Eur J Appl Sci. 2014;6: 11–19. doi:10.5829/idosi.ejas.2014.6.2.8585

6.	Beyene T, Hiko A. Zoonotic metacestodes and associated fi nancial loss from cattle slaughtered at Yabello municipal abattoir ,. Parasite Epidemiol Control. 2019;3: e00096. doi:10.1016/j.parepi.2019.e00096

7.	Haftu B. Study on Prevalence and Economic Significance of Bovine Hydatidosis in Bako Muncipal Abattoir, West Shoa Zone, Oromiya Regional State. J Vet Sci Technol. 2014;5: 1–5. doi:10.4172/2157-7579.1000197

8.	A Melaku A, Lukas B, Bogale B. Cyst Viability, Organ Distribution and Financial Losses due to Hydatidosis in Cattle Slaughtered At Dessie Municipal Abattoir, North-eastern Ethiopia. Vet World. 2012;5: 213–218. doi:10.5455/vetworld.2012.213-218

9.	Kebede N, Mitiku A, Tilahun G. Hydatidosis of slaughtered animals in Bahir Dar Abattoir, Northwestern Ethiopia. Trop Anim Health Prod. 2009;41: 43–50. doi:10.1007/s11250-008-9152-3

10.	Polydorou K. Animal health and economics. Case-study: Echinococcosis with a reference to Cyprus [dogs]. 1981. Available: https://api.semanticscholar.org/CorpusID:201995942

11.	Gebremichael D, Feleke A, Terefe G, Lakew M. Infection rates , risk factors and cyst fertility of hydatid disease in camels in Ayssaita district , Northeastern Ethiopia. Glob Vet. 2013;11: 465–471. doi:10.5829/idosi.gv.2013.11.4.76146

12.	Bekelle T, Gebreyohannes M, Tesfaye A. Identification of pulmonary lesions in slaughtered cattle and associated risk factors , North West Ethiopia. African J Agric Res. 2017;12: 1447–1450. doi:10.5897/AJAR2017.12215

13.	Terefe Y, Addy F, Alemu S, Mackenstedt U, Romig T, Wassermann M. Genetic characterization of Echinococcus species in eastern Ethiopia. Vet Parasitol Reg Stud reports. 2019;17: 100302. doi:10.1016/j.vprsr.2019.100302

14.	Addy F, Alakonya A, Wamae N, Magambo J, Mbae C, Mulinge E, et al. Prevalence and diversity of cystic echinococcosis in livestock in Maasailand, Kenya. Parasitol Res. 2012;111: 2289–2294. doi:10.1007/s00436-012-3082-8

15.	Mbaya H, Magambo J, Njenga S, Zeyhle E, Mbae C, Mulinge E, et al. Echinococcus spp. in central Kenya: a different story. Parasitol Res. 2014;113: 3789–3794. doi:10.1007/s00436-014-4045-z

16.	Nungari L, Mbae C, Gikunju J, Mulinge E, Kaburu T, Zeyhle E, et al. Prevalence and Genotyping of Echinococcus Species from Livestock in Kajiado County, Kenya. Biomed Res Int. 2019;2019: 4798906. doi:10.1155/2019/4798906

17.	Omer RA, Dinkel A, Romig T, Mackenstedt U, Elnahas AA, Aradaib IE, et al. A molecular survey of cystic echinococcosis in Sudan. Vet Parasitol. 2010;169: 340–346. doi:10.1016/j.vetpar.2010.01.004
